# Cholesterol metabolism: a positive target to revoke the function of exhausted CAR-NK cells in tumor microenvironment

**DOI:** 10.3389/fphar.2024.1440869

**Published:** 2024-09-16

**Authors:** Jingfeng Liu

**Affiliations:** ^1^ Shenzhen Key Laboratory of Immunity and Inflammatory Diseases, Peking University Shenzhen Hospital, Shenzhen, China; ^2^ Department of Rheumatism and Immunology, Peking University Shenzhen Hospital, Shenzhen, China; ^3^ Institute of Biomedicine and Biotechnology, Shenzhen Institute of Advanced Technology, Chinese Academy of Science, Shenzhen, China

**Keywords:** CAR (chimeric antigen receptor), NK cell, cholesterol, tumor microenvironment, metabolism

## Introduction

The tumor immune suppressive microenvironment (TIME) within solid tumors is one of the primary reasons why Chimeric antigen receptors (CAR) immune cell therapies fail to exert their tumor-killing effects (Pan et al., 2022; Maalej et al., 2023). Tumor cells functionally shape this suppressive microenvironment by secreting various cytokines, chemokines, or through cell-cell contact regulation, thereby reprogramming surrounding cells to play decisive roles in tumor survival and progression (Mempel et al., 2024). In this process, immune cells act as key regulatory elements. Studies have shown that under the influence of TIME, infiltrating T cells can differentiate into suppressive regulatory T cells (Treg cells), macrophages can shift from an inflammatory M1 phenotype to a suppressive M2 phenotype, and even NK cells can upregulate their inhibitory receptors (Terrén et al., 2019; Nakamura and Smyth, 2020; Allen et al., 2022). These changes induce immune evasion by the tumor, thereby promoting tumor progression and metastasis. Recently, it was raised that active cholesterol metabolism is one of the hallmark characteristics of cancer. Increasing evidence suggests that cholesterol regulates tumor cell proliferation, invasion, and metastasis (Huang et al., 2020). Elevated cholesterol levels often indicate a poor prognosis for tumors. Studies have found that breast cancer patients have cholesterol-derived carcinogenic metabolites that can bind to glucocorticoid receptors, subsequently promoting tumor growth (Voisin et al., 2017). Related research also indicates that blocking cholesterol synthesis metabolism in liver cancer, colorectal cancer, and melanoma can effectively inhibit tumor growth and metastasis, highlighting cholesterol metabolism as a potential therapeutic target for tumors (Gao et al., 2021; Wen et al., 2018). In addition to regulating tumor growth, cholesterol metabolism also influences the antitumor effects of infiltrating immune cells in the TIME. Studies have shown that increased cholesterol metabolism in tumor cells leads to the exhaustion of infiltrating CD8^+^ T cells and is positively correlated with the expression of inhibitory receptors such as PD-1, TIM-3, and LAG-3 (Ma et al., 2019). In this case, to fully understand the role of cholesterol metabolism within TIME would be helpful for developing CAR based precision tumor therapies.

CAR immune cell therapy is an emerging approach in cancer immunotherapy that, through genetic editing, endows immune cells with the ability to precisely target and kill cells expressing specific tumor antigens, offering new hope for cancer cures (Pan et al., 2022). The CAR-T therapy targeting CD19-positive B-cell leukemia and lymphoma was the first CAR immune cell therapy proven effective in clinical treatment (Park et al., 2018). However, the severe graft-versus-host disease (GVHD) caused by allogeneic T cells necessitates the production of personalized CAR-T products for each patient, which is time-consuming and costly. Additionally, the loss of tumor antigens can lead to CAR-T cells losing their targets, resulting in tumor immune evasion and relapse. Given these limitations, natural killer (NK) cells, as innate immune cells, have a promising future in CAR applications due to their ability to rapidly kill cells lacking Major histocompatibility complex (MHC) class I molecules (such as tumor or mutated cells). Besides utilizing CAR activation pathways, NK cells can also exert their cytotoxic effects through their natural membrane receptors (natural cytotoxicity receptors, NCRs, such as NKG2D, NKp46, NKp30, NKp44), thereby reducing the risk of tumor immune evasion and relapse caused by the loss of tumor antigen expression. Furthermore, allogeneic NK cells pose a lower risk of causing GVHD side effects, offering higher safety and the potential to develop universal CAR-NK cells, which could reduce treatment costs (Pan et al., 2022). However, within the TIME, NK or CAR-NK cells often become dysfunctional or exhausted, limiting their therapeutic efficacy. Emerging evidence suggests that cholesterol metabolism plays a pivotal role in modulating the function of NK cells within the TIME.

## Targeting cholesterol metabolism to revoke CAR-NK function

Cholesterol is an essential component of cell membranes and is involved in various cellular processes, including signaling and membrane fluidity. However, excessive cholesterol accumulation can have detrimental effects on immune cells, including NK cells. According to a study published by Huang et al. (2020), cholesterol accumulation in NK cells impairs their function by disrupting membrane lipid rafts, which are crucial for signal transduction. In the TIME, cancer cells and associated stromal cells produce high levels of cholesterol and its derivatives, leading to an environment rich in cholesterol. This excessive cholesterol impairs NK cell function through several mechanisms: 1. Disruption of Lipid Rafts: Cholesterol accumulation disrupts lipid rafts, which are specialized membrane microdomains essential for clustering receptors and signaling molecules. This disruption impairs the activation signals necessary for NK cell cytotoxic function. To some extent, CAR expression on CAR-NK cells may lose their functions due to the membrane disruption. 2. Inhibition of Cytokine Production: Elevated cholesterol levels interfere with the production of key cytokines such as Interferon-γ (IFN-γ) and Tumor Necrosis Factor-α (TNF-α), which are critical for NK cell-mediated tumor cell killing. 3. Metabolic Reprogramming: High cholesterol levels induce metabolic reprogramming in NK cells, leading to a shift from oxidative phosphorylation to glycolysis, which is less efficient for sustained cytotoxic activity. Furthermore, cholesterol accumulation in NK cells also impacts gene expression, further contributing to their dysfunction (Kobayashi et al., 2020). One key regulatory mechanism involves the upregulation of the gene Sterol Regulatory Element-Binding Protein 2 (SREBP2), a transcription factor that regulates cholesterol biosynthesis and uptake. Increased SREBP2 activity leads to higher expression of genes involved in cholesterol synthesis and uptake, such as HMG-CoA reductase (HMGCR) and Low-Density Lipoprotein Receptor (LDLR). Recently, a research group identified MEF2C as a master regulator within SREBP2 associated cholesterol metabolism which may be targeted to revoke NK cell immunity (Li et al., 2024). Simultaneously, cholesterol accumulation downregulates the expression of genes involved in NK cell activation and cytotoxicity. For instance, the gene Perforin (PRF1), which encodes a protein crucial for the lytic function of NK cells, is downregulated. Similarly, Granzyme B (GZMB), another critical molecule for inducing apoptosis in target cells, shows reduced expression. These changes collectively impair the ability of NK cells to effectively recognize and kill tumor cells. To counteract the negative effects of cholesterol on NK cell function, several strategies can be employed to inhibit cholesterol metabolism within the TIME, which might rejuvenate exhausted NK cells and restore their cytotoxic functions (Huang et al., 2020). For instance, statins are inhibitors of HMG-CoA reductase, a key enzyme in the cholesterol biosynthesis pathway. By reducing intracellular cholesterol levels, statins can restore lipid raft integrity and improve NK cell signaling and function. Furthermore, Liver X Receptors (LXRs) are nuclear receptors that regulate cholesterol homeostasis. Activation of LXRs promotes cholesterol efflux and reduces intracellular cholesterol accumulation. LXR agonists can thus enhance NK cell functionality by maintaining optimal cholesterol levels. What’s more, Cholesterol Efflux Promoters, agents that promote cholesterol efflux, such as apolipoprotein A-I mimetics, can help reduce intracellular cholesterol levels in NK cells, thereby improving their cytotoxic activity. However, the limitations of targeting cholesterol metabolism within NK cells should not be overlooked. Normal cholesterol metabolism is essential for various biological processes, and the administration of anti-cholesterol metabolism drugs at specific concentrations necessary to restore NK cell function may induce side effects. Furthermore, there are currently limited clinical trials investigating the combination of CAR-NK cells with anti-cholesterol metabolism drugs. Therefore, it is crucial to conduct more trials to evaluate the efficacy and safety of this combination.

## Discussion

In conclusion, targeting cholesterol metabolism represents a promising strategy to rejuvenate exhausted CAR-NK cells within the TIME. By understanding the mechanisms through which cholesterol accumulation impairs NK cell function and employing strategies to inhibit cholesterol metabolism, we can potentially enhance the efficacy of CAR-NK cell-based cancer immunotherapies. Further research is needed to validate these approaches and translate them into clinical practice, offering new hope for patients with difficult-to-treat cancers, like solid tumors.
